# Experiences of navigating anticipations and anxiety among patients having surgery for peripheral nerve tumours

**DOI:** 10.1038/s41598-025-20906-w

**Published:** 2025-10-03

**Authors:** Emanuel Istefan, Alice Giöstad, Ingela Carlsson, Lars B. Dahlin, Erika Nyman

**Affiliations:** 1 Department of Biomedical and Clinical Sciences, Linköping University, Linköping, 581 85 Sweden; 2 Academic Primary Health Care Center, Region Stockholm, Stockholm, Sweden; 3 Division of Family Medicine and Primary Care, Department of Neurobiology, Care Sciences and Society, Karolinska Institutet, Stockholm, Sweden; 4 Department of Translational Medicine – Hand Surgery, Lund University, Malmö, 205 02 Sweden; 5 Department of Hand Surgery, Skåne University Hospital, Malmö, 205 02 Sweden; 6 Department of Hand Surgery, Plastic Surgery and Burns, Linköping University Hospital, Linköping, 581 85 Sweden

**Keywords:** Peripheral nervous system neoplasms, Upper limb, Anxiety, Surgery, Quality of life, CNS cancer, Surgical oncology

## Abstract

Peripheral nerve tumours, most commonly schwannomas and neurofibromas, might present significant challenges by eliciting physical symptoms and emotional distress. We aimed to explore patients’ expectations, perceptions, and experiences regarding their diagnosis and the surgical treatment of a nerve tumour in the upper limb. Fourteen adults (10 women, 4 men; age range 35–85) from two Swedish hand surgery clinics participated in semi-structured interviews. Data saturation was reached. The interviews were transcribed verbatim and analysed following a conventional content analysis approach. Three main categories emerged. *Causal Reasoning and Anticipations* included explanatory thoughts of tumour aetiology, expectations of surgery, and concerns for the future. *Anxiety and Adaptation* comprised psychological strain, but also how to overcome challenges in daily life. *Course of Care* encompassed uncertainty and waiting time, interaction with health care and decision making on whether or not to have surgery. Recognising the interplay between physical symptoms and psychological responses underscores the need for personalised communication and patient-centred strategies, such as clear and empathetic information and shared decision-making, to reduce uncertainty and support individuals facing peripheral nerve tumours in the upper limb.

**Supplementary Information:**

The online version contains supplementary material available at 10.1038/s41598-025-20906-w.

## Introduction

Nerve tumours comprise a heterogeneous group of tumours, schwannomas and neurofibromas being the most common. They may occur as solitary neoplasms or multiple lesions, often with a simultaneous genetic disorder, such as neurofibromatosis type 1 (Morbus Recklinghausen), neurofibromatosis type 2, or schwannomatosis^1–3^. Schwannomas requiring surgery are estimated to occur in 0.62/100 000 inhabitants and year^4^. Schwannomas and neurofibromas are usually slow-growing tumours and tend to cause symptoms such as pain, paraesthesia, and motor dysfunction as well as disability^4–7^. The incidence of malignant transformation in schwannomas is rare^8^.

Surgical intervention is recommended for symptomatic relief of schwannomas and neurofibromas^4,8,9^. However, temporary new symptoms or augmentations of existing problems, such as pain, paraesthesia, and sensory dysfunction, might occur postoperatively, and sometimes become persistent as perioperative strain on the nerve cannot always be avoided^10,11^. Surgery may also be performed for diagnostic purposes, allowing pathoanatomical diagnosis but leading to patients having to face preoperative diagnostic uncertainty. In benign cranial nerve tumours, such as vestibular schwannomas, premorbid psychological symptoms predicted greater postoperative distress, highlighting the relevance of psychological factors in benign tumour surgery contexts^12^.

This qualitative study aims to further develop the understanding of expectations, patient perception, and experiences related to diagnosis and treatment, as well as anticipation concerning the therapy and the ensuing outcomes regarding nerve tumours in the upper limb.

## Methods

### Study design and participants

A qualitative descriptive method with an inductive approach was used to study experiences among patients with peripheral nerve tumours in the upper limb^13,14^. The study included patients for whom surgery was planned or who had undergone surgery for a peripheral nerve tumour. The participants were recruited from two hand surgery departments in Sweden: the Department of Hand Surgery, Plastic Surgery and Burns, Linköping University Hospital, and the Department of Hand Surgery, Skåne University Hospital, Malmö.

Participants over the age of 18 years without any serious mental, cognitive or linguistic impairments were eligible. Treating senior hand surgeons (L.B.D. and E.N.) assessed this during routine outpatient visits to ensure individuals could meaningfully participate in interviews. Fourteen possible participants were identified: eleven who had already undergone tumour excision and three who were awaiting surgery. To maximise heterogeneity, purposive sampling was applied across sex, occupation, and age^15^. Due to the low incidence of peripheral nerve tumours, every eligible individual awaiting surgery, during the study period, was invited to participate. Eight of the participants had received results from pathoanatomical diagnosis (8/11).

### Procedure and ethics

Informed consent was obtained before the participants were interviewed, and was reconfirmed at the outset of each interview. Participants were given time to consider participation and had the opportunity to ask questions or address any concerns both at the time of recruitment as well as before and after the interviews.

A semi-structured interview guide was constructed by all the authors, comprising questions concerning everyday life experiences, anxieties, fears, and self-perceived health regarding the nerve tumour, as well as the contact with and referrals between healthcare providers, psychosocial demands, and support. The interview guide also included prespecified follow-up questions facilitating more considered responses (Appendix 1). The interview guide was revised after one pilot interview in order to clarify and improve the ordering of the questions. As no questions were added or removed following this revision, the pilot interview was included in further qualitative analyses. Analysis and data collection proceeded iteratively. After the 12th interview no new codes emerged, and in total, 14 interviews were performed. Interviews were conducted by the first author (E.I.). Participants were allowed to choose an in-person interview (1/14), an over-the-phone interview (3/14), or an encrypted video communication platform interview (10/14) based on their preferences. One interview was held in English at the interviewee’s initiation; the rest were held in Swedish. All the interviews were recorded and transcribed verbatim, and field notes were also made. The first author (E.I.) transcribed the pilot interview and two others, while the remainder were transcribed by an individual independent of the current research group. The transcripts were checked for accuracy by the first author (E.I.) and any missing words were added. The median interview time was 64 min (IQR [47–96]), ranging from 38 min to 126 min.

The study was planned and conducted in accordance with relevant guidelines and regulations, including the Helsinki Declaration^16^. The Swedish Ethical Review Authority (No 2020 − 01484 0617) approved the study. Due to the rarity of peripheral nerve tumours and the disclosure of the locality of the hand surgery departments, demographic information is intentionally presented at a group level in order to maintain participants’ anonymity.

### Data analysis

The transcribed texts were analysed using conventional content analysis^13,14^. The transcriptions of the interviews were read by all the authors and re-read by the first and second authors (E.I. and A.G.) to gain an overview of the content. Transcript notations or summaries were utilised separately by all authors. The authors E.I. and A.G. used qualitative data analysis software to organise meaning units, codes, subcategories, and categories within the transcripts [Lumivero, Denver, CO (2023) NVivo (Version 1.7.2) www.lumivero.com]. Meaning units and codes were developed by the first and second authors (E.I. and A.G.), initially independently and then later merged after discussions. Sorting and comparing different codes yielded subcategories, which in turn were grouped under categories. Examples of the analysis procedure are presented in Table 1. Categories and subcategories were discussed amongst all authors to ensure that the categories covered all aspects of the text and were related to the aim (investigator triangulation). Lastly, quotations were selected to exemplify identified categories.

**Table 1:** Examples of the analysis procedure.

Raw data transcript	Meaning unit	Code	Subcategory	Category
“But I remember thinking beforehand – how could they possibly remove something that’s sitting on a nerve? It’s just so tiny.”	Surgical intervention on the peripheral nerves could be intricate	Worries regarding surgery	Expectations of surgery and concerns for the future	Causal reasoning and anticipations
“But then it sometimes felt like there was no one nearby who understood your worry either, because no one else has that kind of problem […]. I remember feeling a bit alone in it.”	Nobody around understood my situation, so I felt alone	Alone with one’s problem	Psychological strain	Anxiety and adaptation

Regarding the authors’ preunderstanding: the first author (E.I.) is a PhD student and a medical intern educated in qualitative research; the second author (A.G.) is a resident working in general medicine with experience in conducting qualitative analysis; the third author (I.C.) is an experienced occupational therapist specialising in hand rehabilitation, with extensive experience of qualitative research; the fourth (L.B.D.) and fifth authors (E.N.) are experienced hand surgeons, familiar with or experienced in qualitative research. The participants had not previously been treated by the first, second, or third authors; however, they had been under the care of the fourth or fifth author.

## Results

Fourteen participants were included: ten women and four men, with a mean age of 56 years (range 35–85). The mean time since surgical treatment was six years (range one month to 14 years). Participant occupations were physically demanding in six cases, non-physically demanding in four, and a further four participants were unemployed or retired. Two participants reported a history of a cancer diagnosis, malignant melanoma and breast cancer, respectively.

Three main categories emerged: *Causal Reasoning and Anticipations*, *Anxiety and Adaptation*, and *Course of Care*. An overview of the main categories and their subcategories is presented in Table 2.

**Table 2:** Main categories and subcategories.

Main categories	Subcategories
Causal Reasoning and Anticipations	Explanatory thoughts
	Expectations of surgery and concerns for the future
Anxiety and Adaptation	Psychological strain
	Overcoming challenges in daily life
Course of Care	Uncertainty and the waiting time
	Interaction with healthcare
	Decision to have surgery or not

### Causal reasoning and anticipations

#### Explanatory thoughts

Previous experiences with different health-related issues impacted the participants’ perception of their symptoms and their thoughts in various ways. Several different explanations for the cause of the nerve tumour were suggested. Some hypothesised that certain factors, such as diet or energy, could influence the occurrence of a nerve tumour. Others thought their symptoms could be associated with other conditions, such as increasing age, multiple sclerosis, malignant melanoma, rheumatoid arthritis, and general overproduction of collagen. One participant thought the bump on their finger came from a thorn after gardening. Disappointments due to previous misdiagnoses were mentioned and in one case the situation was explained as a recurrence of carpal tunnel syndrome. Participants who were healthcare professionals themselves (nurse or nursing-assistant) drew on their knowledge, more often looking for benign explanations for their tumours.

D7: “It shouldn’t be there, so I’ll probably have to go and look it up and check it out. But I’m pretty sure it was a ganglion or something else that was the source of the lump.”

A benign explanation could be attributed if the palpable mass was small. Larger nerve tumours were described as alarming, especially if there was simultaneous pain from the affected area, evoking a need to seek healthcare assessment. Some participants were not as specific in their explanations, saying that something just “felt off”. Tumour growth was also a reason for seeking healthcare, either on the participant’s own initiative, or after encouragement from relatives. Tumour growth had the potential to evoke distress, making participants think they might have missed the opportunity to have an easy intervention, and worry that their delay had caused a malignant transformation of the tumour. Some individuals were not concerned about the mass itself, but more about the fact that the tumour emerged from the nerves.

D3: “Yes, the concerns were probably not so much about the psychology of the lump itself in the first place, but I was more like, okay, wait a minute here. Now there is some kind of small tumour mass that originates from the nerve itself.”

Having a previous history of cancer or experience of a relative with a cancer diagnosis could intensify emotion concerning the presence of a tumour. Using the word “tumour” versus “cancer” when delivering the diagnosis was, therefore, considered important.

D2: “And I do know that tumour does not equal cancer and death. I actually know that, but inside it triggers something anyways”.

D10: “Yeah, since they said that it was nothing dangerous, no dangerous cancer, I was calm”.

#### Expectations of surgery and concerns for the future

Different expectations regarding the surgery, subsequent symptoms, and long-term functionality and quality of life were described and discussed. Long-term functioning, in order to be able to provide for and support families or children, was seen as being of great importance, and in some cases was expressed demand, before agreeing on surgery. Others worried about the possibility of symptom augmentation.

D2: “No, it is about how it will develop and how it’s going to go regarding both the insensitivity, numbness in my fingers, and the strength? How, how? And if it (*the tumour*) gets worse quickly, what then? Then I will become a one-armed bandit”.

Individuals also said that since hospital admission was not planned after surgery, the procedure must be minor and therefore not a cause for concern. Hopefulness for complete recovery from the symptoms was also described. Worries were expressed about the technical aspect of the surgery as were concerns that surgical intervention on the peripheral nerves could be intricate and might be associated with a subsequent risk of deteriorating sensory or motor function.

D12: “However, I’m afraid of losing control, […] that’s not really right, rather that I’m not steering things, and that is what I feel a bit has been going on in my hand these last few years. There’s something going on in my hand and it’s out of my control. And that’s a little scary in itself because I think that’s what I’m most afraid of, being in my body and yet not being able to move like me.”

### Anxiety and adaptation

#### Psychological strain

Having a peripheral nerve tumour in the upper limb could cause a variety of different symptoms. To handle the psychological strain, the participants sought support from different sources, with varying effects. Trying to manage the distress, the participants found both alleviating and aggravating factors. After noticing the tumour, they expressed an increased level of self-consciousness and vulnerability.

D1: “Yes, in a way, because when I think about it, it probably did because it also resulted in me becoming a little more of a hypochondriac in a way because then you are a bit more careful or something.”

The view was also expressed that, due to the presence of the tumour, the individual affected no longer felt they were invulnerable to rare conditions, prompting fears that if they could develop such an uncommon illness, they might also be at risk of getting other diseases. The tumour also reminded participants of their mortality. In contrast, others developed no change in perceived health-related vulnerability. Emotional distress, often triggered by thoughts of abnormality, malignancy, metastasis, or mortality, was associated with a reoccurrence of previous avoidance habits, such as comfort eating. After becoming aware of their nerve tumour, patient delay was also expressed as the participants realising they needed medical assessment, but hesitating to seek help right away because of denial.

D1: “I thought I would seek medical advice when I discovered it, but it was sort of a case of putting my head in the sand.”

Experiencing a lack of understanding from people in your surroundings, or seeing oneself as a burden to one’s family were also described as factors contributing to psychological strain and experiences of abnormality. A feeling of loneliness and that no one understood what they were going through was also voiced. Others found great support from their partners, relatives, friends, or colleagues. A sense of community was also found on dedicated pages on social media. Another participant with a malignant peripheral nerve tumour had a hard time accepting the diagnosis, resulting in a conflicting self-image. The same individual reported dark thoughts immediately after surgery together with concerns about the remaining loss of function.

D5: “I remember that I felt pretty down. I recall my diary enteries, so I know I thought it was tough […]. After the first surgery, I don’t remember it being that tough, but I do remember having pretty dark thoughts then. After the second surgery, well, I was simply a bit depressed. And not just a little depressed either. I was probably quite depressed”.

Utilising humour or downplaying the severity of the situation through comparisons with other diseases were strategies mentioned for managing the stressful situation. Acceptance was also described as a way of dealing with psychological strain where participants accepted risks and uncertainty. Hoping for a gradual recovery helped some to accept their postoperative symptoms.

D14: “It is a kind of […] you try to joke about it a bit, even if you are a bit scared about what might happen. You try to laugh it off”.

#### Overcoming challenges in daily life

Having a peripheral nerve tumour in the upper limb caused a great variety of symptoms and challenges that participants handled in their own way. Participants highlighted an increased need to adjust or change their grip, as well as use the other hand or tools to perform daily tasks. Switching to automatic cars due to an inability to handle manual transmission was one solution mentioned.

D2: “I always have a polygrip plier in the kitchen nowadays, because that’s what I use when I can’t get the lids off; like the top of the milk cartons.”

Other changes included heightened awareness in public situations; for instance, experiencing difficulty relaxing on public transport, deliberately keeping the arm clear to prevent accidental contact with the painful mass.

D13: “So I started like to try to hide my, my arm whenever I, I had a lot of people around me, so nobody grabbed me with their hand or something, you know.”

Although participants in some cases became accustomed to their discomfort over time, the symptoms and conditions continued to impact social and recreational activities, with the tumour restricting the pursuit of certain sports. As a consequence, participants switched to more suitable activities instead.

D4: “I used to play handball, and such. I can’t do that any more. I can’t play badminton. […] But there are other activities, going fishing for example. That works great”.

Working life was also impacted by the nerve tumour. Participants described the need to take sick leave as being particularly burdensome, both because of the effect on the household economy, as well as the decreasing sense of meaningful context from being on sick leave. One participant with a history of social bullying due to the visibility of the tumours explained choosing a more readily available career to avoid a feeling of exclusion.

D8: “When I am on sick leave, I lose a lot of money. It is important for everything. I need to save a bit [in advance …] you know. My finances aren’t working so well.”

### Course of care

#### Uncertainty and the waiting time

Uncertainty during care was described as worrying, demanding, and distressing. The participants expressed uncertainty at several stages of care with multiple waiting periods, awaiting results from diagnostic examinations, and time lapses between referrals.

D1: “I think I went and had the first ultrasound in April, I think it was. Then I had the MRI in the summer and got the results in August. So, there were a few months of uncertainty there.”

The existing uncertainty regarding the tumour was amplified by a perceived inefficiency of the healthcare apparatus. There was a desire for clear information and guidance on where to seek further professional support during this period.

D1: “I would have wanted more information about what was going to happen, you know. […] not really knowing who to turn to if you have questions or concerns”.

A time frame was requested to provide advance notice, to set expectations and to reduce uncertainty.

D3: “I can accept a certain level of waiting, of course, [but] what does the plan look like? […] What I’m interested in is: what are we talking about here? Are we talking four months, or are we talking two years?”

The waiting time on the actual day of surgery was especially burdensome for some, who described a fear of being forgotten in the hours before the operation.

#### Interaction with healthcare

The importance of receiving, understanding, and valuing information from healthcare providers was emphasised. Communication was highlighted both as a challenge and a crucial element in the patient-doctor relationship. The participants described various difficulties as well as positive instances of the interaction with healthcare. Patient participation, as well as ideas about optimal patient involvement with their healthcare, were emphasised.

D12: “[The surgeon] conveyed that to me, and she was also very clear about what she saw in this, and what she wanted to do, and then I also felt very safe with it. I felt that I was actually facing a good doctor who didn’t need to ask me what they should do”.

Just providing information did not always mean that participants understood their condition. Some received answers that they did not understand and ended up searching for information themselves. In contrast, personalised information was appreciated, and participants valued the use of simple, non-medical, language when possible. Processing the verbal information provided by the physician continued after the visit, and sometimes new or unanswered questions arose. Information availability was appreciated by those who wanted to check their medical records digitally and was described as a way in which participants could follow their medical journey. The chance to ask for more information or an explanation from surgeons was appreciated.

D14: “I got the feeling from [the surgeon] that if I wanted more information, he would willingly provide it. […] I gained more inner peace and felt safer. You felt [the surgeon] was, you know, patient-safe. He wanted, you know, to convey the information he had…”.

The participants wanted a varying degree of participation in their care. For some, the authority of healthcare professionals was reassuring, while others wanted to play a more active role in the decision-making. Some found the needle biopsy of the nerve tumour very uncomfortable and in retrospect wished they had discussed the benefits of the examination against the procedural cost of significant discomfort. The desire to be asked about how much they wished to participate included the operating room. One participant was shown a monitor with a digital feed from the microsurgical procedure, under the assumption that they wanted to see it when that was not the case.

A professional’s demeanour, attentive listening, and positive attitude provided participants with a sense of security. The feeling of being seen and taken seriously was mentioned as an example of a professional approach that was appreciated. One participant expressed the disappointment they felt when a junior doctor in a primary healthcare facility setting, in their view, made up their mind regarding the most likely diagnosis of the patient’s problems, without taking the patient’s symptoms seriously. As the patient’s perspective is often multifactorial and individualistic, one participant emphasised the importance of acknowledging the broader personal context that may influence how patients experience and interpret their condition and treatment:

D12: ” In all of this, maybe you don’t think about all of this when you hear you have a lump in your hand, there are so many other things that affect this particular operation.”

#### Decision to have surgery or not

The decision to undergo surgery was significant and complex for many. Various considerations were reviewed, with avoidance of symptom worsening/progression being a priority for some. The participants theorised that the symptoms could worsen if surgery led to nerve damage as a complication or if surgery was postponed and the tumour grew, further affecting the nerve. This fear of aggravating the nerve was clearly expressed by one participant who said, D10: “Yes, I was thinking it could lead to more nerve damage. […] So that it could get even worse”. Surgical treatment to remove a nerve tumour was perceived as complicated because the tumour involved the nervous system, which was perceived as possibly intricate and generally more difficult.

D11: “Before I thought, how can they possibly remove something that is attached to a nerve? It is just so tiny”.

D9: “So, it’s, it’s kind of a balancing act there, you know. But it doesn’t really bother me that much at all. I sleep well at night, and it’s like nothing. I don’t have any pain when I’m working”.

Repeated general anaesthesia could be thought of as burdening the body and was another contributory reason for postponing surgery. Concurrent plans to have children, obligations toward existing children, and household finances were other reasons given for delaying surgery.

D1: “At first I just felt ouch, that felt a bit strange […]. And then, it became more and more irritated, and of course you get worried […] so then I sought medical advice for it [… and …] I found out that it wasn’t anything dangerous or that, and then I was able to decide for myself whether it was something I wanted to have removed right then or not. And just then there was a lot going on in my life like so, ‘okay, I’ll postpone it a bit, I don’t really have time to be on sick leave right now’, […] and then it was like ‘we’ll deal with it later’. […] I’ve more or less learned to live with it”.

Patient autonomy was considered very important. One participant highlighted pressure from relatives, who also decided that the patient would undergo surgery. In retrospect, the individual regretted the surgery due to permanent postoperative symptoms, blaming themselves and the relative in question.

D6: “I did not have any trouble. I did not have any pain, nothing. It was my daughter who forced me to have the surgery. […] I did not care about the lump. I did not care about anything. I didn’t want this operation. I didn’t. But my daughter forced me.”

Shared decision-making between patient and healthcare professional was much appreciated. Those who initially preferred to postpone surgery were worried about being overruled by an eager surgeon. Some were concerned that the prospective surgeon would decide for them. In contrast, other individuals felt relieved not to have to make this significant decision themselves and appreciated the surgeon providing a clear recommendation.

D10: “[The surgeons] wondered if I wanted to have the operation, but then I thought like this: maybe it gets even worse if I decide on surgery, so I have not done it, and [the surgeons] thought that it was a good decision. […] They agreed with me that [the surgery] could make it worse. You never know, […], but maybe”.

## Discussion

Patients with nerve tumours in the upper limb face unique challenges that significantly impact their daily lives, affecting both their physical health and psychological well-being. This study explores their experiences from the initial discovery of a nerve tumour in the upper limb through navigating the healthcare system, receiving the suspected diagnosis, and undergoing the surgical treatment. This study also describes how patients contextualise their symptoms and anxieties related to the presence of a palpable tumour in the peripheral nerve.

Our research identified three main categories: *Causal Reasoning and Anticipations*, *Anxiety and Adaptation*, and *Course of Care*, each comprising two or more subcategories. Past life experiences and personal beliefs significantly shaped participants’ perceptions of and explanations for their symptoms. Living with a nerve tumour involved considerable psychological strain and emotional challenges, including heightened levels of anxiety and stress, and feelings of vulnerability. These findings resonate with observations presented by Jensen et al. of individuals with neurofibromatosis type 1 associated plexiform neurofibromas, who similarly experienced limitations in social participation and leisure activities, physical function, and pain, as well as initial tendencies toward avoidance and thoughts of denial^17^.

Participants’ accounts reflected core elements of the Leventhal’s Common-Sense Model (CSM) for illness perception^18^. The understanding of the diagnosis varied among participants who attributed the causal nature of their tumours to environmental or self-inflicted (e.g. due to diet or lifestyle) factors, or association with other diseases. The causal reasoning and anticipations seem to be individualistic and are possibly influenced by previous life experiences. The perception of the vulnerability of the peripheral nervous system, an association between tumour and malignancy, and previous experiences involving oncological diseases in relatives were especially pronounced experiences.

Participants’ expectations of surgery varied greatly. Surgeons should recognise that individual beliefs differ and that circumstances may be interpreted in diverse ways: some participants viewed the surgery as being less complex because there was no planned hospital admission, while others felt anxious because their condition and the surgery involved the peripheral nervous system. Decision-making style, the patients’ trust in the physician, an understanding of surgery as well as past experiences have previously been highlighted as patient-related factors that affect the decision to have surgery in patients with vestibular schwannomas^19^. Effective communication lays the foundation for trust. Building on this trust, shared decision-making can then empower patients without overwhelming them^19,20^. In line with previous research, participants in our study highlighted shared decision-making as an important facilitator for communication between healthcare providers and patients, promoting patient involvement in their own health, and a holistic approach improving autonomy^21^. Shared decision-making is also reported to increase patient satisfaction, reduce anxiety, and improve trust in the physician^19,22^.

Noticing a tumour could prompt increased self-consciousness and self-perceived vulnerability in participants and could introduce fears of impairment or mortality, constituting a health threat consistent with Leventhal’s CSM for illness perception^18^. These effects were individualistic as not all participants reported psychological strain, but amongst those who did, responders appeared to reflect coping processes in line with Leventhal’s CSM, where seeking support and finding acceptance were reported as alleviating factors. The participants’ social network was particularly important and multifaceted as it was described as a source of support that brought relief, or as a constellation where feelings of being misunderstood originated, or as a source of hidden pressure to undergo surgical treatment.

When the peripheral nerve tumour interfered with the participants’ ability to work, the sense of meaningful context could be negatively affected. Acceptance and solution orientation with adjustments in activities, by additional use of tools for example, were sometimes reported, but other physical or social activities were no longer feasible due to the peripheral nerve tumour. Pruijn et al. reported that patients with vestibular schwannomas also had difficulties participating in physical and social activities, and that participants primarily preferred improvement in outcomes regarding their specific limiting symptoms^23^. In contrast, participants in our study also focused on carefully considering waiting for surgery because of possibly deteriorating symptoms.

Uncertainty throughout the care process was a source of distress. Participants emphasised the need for clear, consistent information and defined time frames to better manage expectations, especially among those less familiar with the healthcare system. Perceived inefficiencies and lack of transparency further contributed to feelings of concern and frustration. Previous recommendations focusing on healthcare provider communication to improve illness uncertainty for patients with advanced cancer, also support the reported desire for general clarity in communication to reduce potential distrust^24,25^. However, discussing scientific uncertainties regarding the treatment might negatively affect patients’ satisfaction with their decision^26^.

Communication was described as a crucial element in the relationship with the healthcare provider and was sometimes a challenge. Providing them with information did not always result in participants understanding their condition. Studies of patient-surgeon consultations highlight the importance of ensuring that patients feel comfortable during the consultation and have the opportunity to present their problems in their own words^27^. Exploring the patients’ ideas, concerns, and expectations during the consultation can both enhance the consultation and promote a more inclusive and effective healthcare system^28^. The individuals’ participation in their care was not necessarily appreciated and variations in personal preference were reported. As patients’ perspectives are multifaceted, a holistic framework is needed to take into account aspects that are important in the participants’ view. On the other hand, participants sought information about how they could improve their symptoms, thereby assuming a degree of responsibility for their condition while simultaneously taking greater control over their health.

### Methodological considerations

In qualitative research, findings are evaluated in terms of trustworthiness, which includes establishing credibility, dependability, confirmability, and transferability^14,29^. In this study, in-depth interviews were conducted by an independent researcher, not involved in the surgical treatment, thereby enhancing dependability. Credibility was reinforced through investigator triangulation, with cross-verification of interview data and coding processes. The data analysis involved a structured examination of the transcripts following the principles of qualitative content analysis. The interviewer’s medical background and the surgeons’ involvement in recruitment may have influenced participants’ responses to appear compliant or resilient, introducing potential social desirability bias. To mitigate this, the interviewer clarified their independence from the clinical management, and invited critical feedback during interviews. The analytic stance was primarily descriptive with low degree of interpretation. To minimise the risk of overinterpretation, a deeper latent analysis was not pursued given the modest sample size and rarity of these tumours.

Confirmability was addressed by presenting analytic findings alongside illustrative participant quotations. Throughout the interviews, member checks, meaning participants consistently had the opportunity to adjust and verify their responses during the interview process through repeated summaries, were implemented thereby increasing the credibility and trustworthiness of the research findings. Because some accounts involved recollection of prior events, recall bias is a potential concern. However, participants frequently provided strong and vivid descriptions of specific episodes and care interactions, which we believe helps mitigate this risk. In addition, the use of an independent researcher and methodological triangulation contributed to minimising potential biases, ensuring the authenticity and neutrality of the findings.

While these methodological strategies enhance the robustness and validity of the conclusions, the transferability of the findings is limited by the small sample size, a reflection of the general rarity of peripheral nerve tumours, and the inclusion of both benign and malignant nerve tumours in the upper limb.

This study highlights the multifaceted experiences of patients with peripheral nerve tumours in the upper limb, revealing significant psychological and emotional challenges alongside physical effects. Participants’ expectations of surgery varied greatly, shaped by personal beliefs, life experiences, and misconceptions about malignancy. The findings underscore the need for clear, empathetic communication and shared decision-making to address uncertainty and foster trust. A person-centred approach that incorporates patients’ fears, beliefs, and social contexts is crucial if both care quality and patient experience are to be improved.

### Practical implications for healthcare practice

Implementing effective communication strategies with a holistic framework may help identify and address communication gaps; an essential step in improving patient-provider interactions. One important practical consideration concerns the management of waiting periods for diagnostic results and surgical intervention. Some participants described these intervals as particularly distressing, with prolonged uncertainty exacerbating feelings of vulnerability and distrust towards the healthcare system. Setting clear expectations regarding timelines could alleviate anxiety for some patients and foster a stronger therapeutic alliance.

The diversity in participant attitudes toward the decision-making process underscores the complexity of navigating personal autonomy. While some welcome professional guidance, others fear losing control over their health choices. This highlights the need for healthcare professionals to engage in open, empathetic communication, ensuring that patients feel both supported and empowered when facing significant medical decisions. Shared decision-making plays a crucial role in this context.

Additionally, early identification and assessment of mental health needs, incorporating psychological support earlier when needed, could alleviate the emotional burdens some patients face. As this study underscores the individuality in the perception of the peripheral nerve tumour and subsequent surgery, means that the personalised patient consultation should incorporate patients’ preconceptions, fears, and expectations concerning their specific condition, regardless of its character and severity. The consultation should also cover potential treatment, focusing on preconceived ideas about tumours involving the peripheral nervous system, surgical potential and limitations in the system, and probing the potential association between tumour and cancer.

## Supplementary Information

Below is the link to the electronic supplementary material.

Appendix 1

## Data Availability

Public access to the data is restricted by the Swedish Authorities (Public Access to Information and Secrecy Act; https://www.government.se/information-material/2009/09/public-access-to-information-and-secrecy-act but data can be made available for researchers after a special review that includes approval of the research project by both an Ethics Committee and the authorities’ data safety committees (http:/www.etikprovningsmyndigheten.se/en). Correspondence and requests for materials should be addressed to E.N.
