# Supplementary material for: Experiences of navigating anticipations and anxiety among patients having surgery for peripheral nerve tumours
Source: Sci Rep. 2025 Oct 3;15:34474. doi: 10.1038/s41598-025-20906-w (PMC12494883; doi:10.1038/s41598-025-20906-w)
Supplement: Supplementary file 1 — Appendix 1 [file 41598_2025_20906_MOESM1_ESM.pdf]

Appendix 1.
Interview Guide

Opening questions

- Which arm/hand was operated on?
- Are you currently working? If yes, what do you do?
- Do you have a family?
- What do you do in your free time?

The Nerve Tumour

- Tell me about when you first discovered your lump
- Can you describe your experiences with having a lump?
- Does anyone in your family or among your friends have experience with something similar?
- Have you experienced any anxiety or worry related to your lump? If yes, how would you describe it?
- Have you had any fears or concerns related to your lump?
    - Are there other specific worries you'd like to mention related to your lump?
- Has your view of your body and health changed since discovering your lump? If yes, in what way?

Care Process

- How did you experience your initial contact with healthcare?
    - What type of information did you receive? How did you perceive this information?
      How did you experience the support provided?
      Did it increase your worry or reassure you?
- Tell me about your experience regarding the information you received about your lump
    - What were your initial thoughts and feelings when you first learned about the suspected diagnosis?
    - What were your thoughts and feelings when you received the results from the microscopic examination of your lump?
- How did you experience the period between your healthcare visits and the surgery?
- How did you experience the day of surgery?
- How did you perceive the information and care you received on the day of surgery?
- How did you experience the time following surgery?
    - Short-term and long-term experiences.
- Are you dissatisfied with anything regarding your care and/or surgery and its outcome?
- Is there anything specific you are particularly satisfied with regarding your care and/or surgery and its outcome?
- Do you have any advice for healthcare providers working with this type of diagnosis?

Psychosocial Support

- Have you personally sought any support for your thoughts or worries related to your lump? If yes, what type of support was it (family, friends, professional support)?
- How did you experience this support?
- How has your lump affected your relationships (family, friends, partner)?
- What coping strategies have you used, and which have been effective in managing your thoughts or worries?

Tumour Disease

- Do you have previous experience with other lumps or any other tumour diseases? If yes, which ones?
- Do you feel your past experiences with other tumour diseases have influenced how you manage thoughts, feelings, worries, or fears related to your nerve tumour? If yes, in what way? More/Less?
- How would you describe yourself and your personality?
    - Do you perceive yourself as an anxious person?
- Is there a specific event in your life that might have impacted you or your emotions?

Closing

- Is there anything else you'd like to discuss that we haven't already covered?

Follow-up Questions

- Could you describe that in more detail?
- How do/did you think about that?
- How did you experience that?
- How did you feel about that?
- How did you handle that?
